# Evolutionary Understanding of Aquaporin Transport System in the Basal Eudicot Model Species *Aquilegia coerulea*

**DOI:** 10.3390/plants9060799

**Published:** 2020-06-26

**Authors:** Shweta Singh, Vacha Bhatt, Virender Kumar, Surbhi Kumawat, Praveen Khatri, Pankaj Singla, S.M. Shivaraj, Altaf Nadaf, Rupesh Deshmukh, Tilak Raj Sharma, Humira Sonah

**Affiliations:** 1 National Agri-Food Biotechnology Institute (NABI), Mohali Punjab 140306, India; s24shweta@gmail.com (S.S.); vvs.kumar15@gmail.com (V.K.); surbhikumawat002@gmail.com (S.K.); p.khatri2712@gmail.com (P.K.); pankajsingla2614@gmail.com (P.S.); sraj100@gmail.com (S.M.S.); rupesh0deshmukh@gmail.com (R.D.); 2 Department of Botany, Savitribai Phule Pune University, Pune, Maharashtra 411007, India; vacha.biotech@gmail.com (V.B.); nadaf.altaf@gmail.com (A.N.); 3 Department of Biotechnology, Panjab University, Chandigarh 160014, India; 4 Division of Crop Science, Indian Council of Agricultural Research, Krishi Bhavan, New Delhi 110001, India

**Keywords:** *Aquilegia*, aquaporins, bioinformatics, NPA motifs, transporter, transcriptomics, floral development

## Abstract

Aquaporins (AQPs) play a pivotal role in the cellular transport of water and many other small solutes, influencing many physiological and developmental processes in plants. In the present study, extensive bioinformatics analysis of AQPs was performed in *Aquilegia coerulea* L., a model species belonging to basal eudicots, with a particular focus on understanding the AQPs role in the developing petal nectar spur. A total of 29 AQPs were identified in *Aquilegia*, and their phylogenetic analysis performed with previously reported AQPs from rice, poplar and Arabidopsis depicted five distinct subfamilies of AQPs. Interestingly, comparative analysis revealed the loss of an uncharacterized intrinsic protein II (XIP-II) group in *Aquilegia.* The absence of the entire XIP subfamily has been reported in several previous studies, however, the loss of a single clade within the XIP family has not been characterized. Furthermore, protein structure analysis of AQPs was performed to understand pore diversity, which is helpful for the prediction of solute specificity. Similarly, an AQP AqcNIP2-1 was identified in *Aquilegia*, predicted as a silicon influx transporter based on the presence of features such as the G-S-G-R aromatic arginine selectivity filter, the spacing between asparagine-proline-alanine (NPA) motifs and pore morphology. RNA-seq analysis showed a high expression of tonoplast intrinsic proteins (TIPs) and plasma membrane intrinsic proteins (PIPs) in the developing petal spur. The results presented here will be helpful in understanding the AQP evolution in *Aquilegia* and their expression regulation, particularly during floral development.

## 1. Introduction

Aquaporins (AQPs) are pore-forming membrane proteins that belong to the major intrinsic protein (MIP) family. Aquaporins facilitate selective transport of water and many other small solutes across the biological membranes. The small solutes transported through AQPs include urea; CO_2_; H_2_O_2_; and metalloids such as silicon (Si), boron (B), and germanium (Ge) [1,2]. Aquaporins are present in almost all eukaryotes and prokaryotes [3,4,5]. These proteins were first reported in the erythrocytes of mammals by Peter Agre and his colleagues in the early 1990s [6,7]. The discovery of AQPs paved the way to enhance our understanding of cellular transport systems in animals as well as in plants [1,8]. Being a principal component of the cellular transport system, AQPs are highly crucial for biological functions, and are found to be linked with various physiological processes [9,10,11].

The whole genome sequence information publicly available for diverse animal and plant species has expedited the identification and classification of AQPs [12]. Higher plants have a larger number of AQPs when compared to animals, ranging from 35 in Arabidopsis to as high as 120 in canola [1,13]. The high number of AQPs in plants is most probably due to a higher ploidy level. Several important features of AQPs related to their distribution, genetic organization, evolution, and conserved motifs involved in solute specificity and transport are well characterized [12]. Plant AQPs are generally categorized into five major subfamilies based on their phylogenetic distribution: plasma membrane intrinsic proteins (PIP), nodulin 26-like intrinsic proteins (NIPs), tonoplast intrinsic proteins (TIPs), small intrinsic proteins (SIPs), and uncharacterized intrinsic proteins (XIPs) [1,14]. Genome-wide studies have identified two more subfamilies in non-vascular plant species, hybrid intrinsic proteins (HIPs) and GlpF-like intrinsic proteins (GIPs), suggesting the loss of these lineages in vascular plants [1]. Similarly, the loss of entire XIP subfamily has been observed in monocots and some of the dicots, like in many species belonging to Brassicaceae [13,15].

Identification of the conserved features of AQP protein sequences helps to depict their role of solute specificity [1,16]. Furthermore, the availability of transcriptomics and proteomics data provides a broad base to study the functional aspects of AQPs, thereby expanding the understanding of the AQP transport system [12,17,18]. The enormous amount of data generated by omics approaches has helped to reveal many aspects related to AQP evolution, protein structural and functional dynamics, and pore morphology [12,19,20,21].

The structure of AQPs studied in different plant and animal systems has been well exploited to understand the intricate mechanism of solute permeability, specificity, and selectivity [21,22]. Aquaporins have highly conserved characteristic hourglass-like structures formed with six transmembrane (TM) helices and two half TM helices, each of them harboring an asparagine-proline-alanine (NPA) motif. The NPA motifs form one of the two constrictions present in the center of the AQP pore that regulates the selective transport of solutes. The second constriction is formed primarily from four amino acid residues known as the aromatic arginine (ar/R) selectivity filter (SF) [12]. Another conserved feature known as Froger’s residues found to have a significant role in solute transport through bacterial aquaporins has also been reported [12]. Recently, the spacing between two of the NPA motifs was identified as an essential feature determining the solute specificity of AQPs transporting Si in plants [1].

*Aquilegia coerulea* is the member of the eudicot order *Ranunculales*, phylogenetically equidistant between the model plant species *Arabidopsis* and rice [23]. The characteristic petal nectar spurs in *Aquilegia* maintain reproductive isolation between species by specific pollinator interactions [24]. A broad range of phenotypic variation in *Aquilegia* has been observed across different geographic locations, particularly in respect to flower color and the size of different floral organs, reflecting adaptation to different pollinators [24]. *Aquilegia*’s position as an early-diverging eudicot means that the model system can be used to study the evolutionary divergence of monocots and dicots. *Aquilegia*, as a model plant, has been used extensively for numerous ecological and evolutionary studies [23,25,26,27,28,29]. In addition, many species of the genus are extensively inter-fertile and have been used to produce fertile hybrids, making the genetic dissection of this diversity plausible [25].

Considering its importance as a model species, identification of the key genes controlling oriented cell division, cell elongation, and regulating water balance in floral organs is of interest. Aquaporins are known to play a major role in numerous physiological processes vital for flower development, such as stomatal movement, photosynthesis, petal movement [20,30,31,32], maintenance of cell turgor [33], and cell elongation [30]. Additionally, the transcriptome profiling of AQPs conducted in several plant species has revealed tissue, growth stage, and environmental conditions of specific expression [13,20,31,34]. However, the RNAseq approach has limitations due to inadequate correlation between level of transcription and protein abundance. In this regard, the present study was aimed to characterize the AQP gene family in *Aquilegia* to understand their evolution and gene expression dynamics during the flower development. The aim of the present study was to understand the evolution of AQPs in the *Aquilegia* genome and subsequently study the expression profiles, gene structure, protein motifs, substrate specificity, and pore morphology of AQPs using computational tools.

## 2. Materials and Methods

### 2.1. Genome-Wide Identification of Aquaporins in Aquilegia coerulea

The *Aquilegia coerulea* genome v3.1 was retrieved from the Phytozome database (www.phytozome.net) [35]. A local database of protein sequences of *Aquilegia* was created using BLAST utilities provided in the Bioedit software package Version 7.0.9.0 [36]. The AQP genes were identified with BLASTp search performed using known AQPs from rice, Arabidopsis, and poplar as query sequences. An *e*-value of 10^−5^ and a bit-score greater than 100 were used as a cut-off to identify significant matches. Aquaporin homologs identified with these criteria were used for further analysis.

### 2.2. Classification and Phylogeny of Aquilegia coerulea Aquaporins

Multiple sequence alignment of AQPs was performed using the CLUSTALW tool implemented in the MEGA7 software suite [37]. A phylogenetic tree of AQPs was generated using the maximum likelihood (ML) method, and 1000-bootstraps was performed to measure the stability of branch nodes in the ML tree. The classification of AQP subgroups was performed in accordance with the nomenclature of known AQPs from rice, Arabidopsis, and poplar, which were used as a query. A combined phylogenetic tree of AQPs from Aquilegia, Arabidopsis, rice, and poplar was also constructed. Phylogenetic tree of plant orders was generated based on National Center for Biotechnology Information (NCBI) taxonomy using the online server phyloT (http://phylot.biobyte.de/).

### 2.3. Gene Structure and Conserved Motif Analysis of Aquaporins in Aquilegia coerulea

Conserved domains for Aquilegia AQPs were identified by using the batch mode of NCBI’s Conserved Domain Database (CDD www.ncbi.nlm.nih.gov/Structure/cdd/cdd.shtml). All the known features of AQPs were identified using Microsoft excel utilities and protein sequence alignments generated with MEGA7. Aquaporins with a single or missing NPA motif were manually curated or by using the blastp search in NCBI. The identified AQPs were further screened with TMHMM, SOSUI, and TOPCONS software tools (www.cbs.dtu.dk; http://bp.nuap.nagoya-u.ac.jp) to identify transmembrane (TM) domains [38]. All the AQPs have two half TM helix which make it difficult to correctly predict the TM domains. Therefore, three different softwares were used to confirm the TM domain prediction and, subsequently, homology-based tertiary protein structure modeling was also used to confirm the TM domains.

Conserved motifs in the AQP proteins were identified using the ‘Multiple Expression Motifs for Motif Elicitation’ (MEME), an online accessible software program [39]. The default settings (minimum width 6 and maximum width of 50 amino acid motifs) were used for the initial MEME scan. The final output of MEME was manually examined.

### 2.4. Identification of Subcellular Localization and Structural Characterizations of Aquilegia Aquaporins

The subcellular location of the AQP proteins was predicted by using three different online servers, namely, the Targetp1.1 server (http://www.cbs.dtu.dk/services/TargetP/) [40], Wolfpsort (https://wolfpsort.hgc.jp/) [41] and the subcellular localization predictive system (Cello, http://cello.life.nctu.edu.tw/) [42].

The gene structure of *Aquilegia* AQPs was visualized using the Gene Structure Display Server (GSDS) (http://gsds.cbi.pku.edu.cn/) [43]. The chromosomal locations in the *Aquilegia* genome were assigned to each AQP genes and subsequently visualized using Mapchart software [44].

### 2.5. Protein Structure Prediction

The 2D protein structures of AQPs were visualized with an open-source web tool, Protter (http://wlab.ethz.ch/protter/start/), [45] using the TM domain information confirmed with three different software tools. Homology-based protein tertiary structures of all the *Aquilegia* AQPs were predicted using the Phyre2 protein-modelling server (www.sbg.bio.ic.ac.uk/*phyre2) [46]. The results obtained in the form of PDB files were uploaded to the Mole2.5 server to predict all possible transmembrane pores, channels, and pore-lining residues (https://mole.upol.cz/) [47]. Pore morphology was also studied using an online accessible web server, PoreWalker (http://www.ebi.ac.uk/thornton-srv/software/PoreWalker/) [48].

### 2.6. RNA-Seq Data Analysis

Gene expression of all the identified AQPs in the *Aquilegia* genome was evaluated by using previously published RNA-seq data [29]. The distal 0.5 mm of flower petal spur cups and blades at three stages of development (1 mm, 3 mm, and 6–7 mm spur length) were used for RNAseq analysis [30]. The transcript abundance was normalized by the FPKM (fragments per kilobase of transcript per million mapped reads) using cufflink tools. The normalized expression data for all *Aquilegia* AQPs were analyzed further with the MultiExperiment Viewer (MeV_4-9-0) software tool (http://www.tm4.org/mev.html). RNA-Seq data for different petal and spur tissues and conditions are deposited in the public database under the BioProject ID PRJNA270946 and previously described in Yant, Collani, Puzey, Levy and Kramer [29].

## 3. Results

### 3.1. Genome-Wide Identification, Classification and Phylogenetic Distribution of Aquaporins in Aquilegia coerulea

Initially, a total of 35 AQPs were identified in the *Aquilegia* genome based on a homology search performed using known AQPs from rice, Arabidopsis, and poplar. Subsequently, 29 AQP genes were sorted after removing alternate transcripts of the same gene and truncated genes (Table 1). A similarity search performed using the conserved domain database (CDD) tool categorized all 29 AQPs as members of the MIP family and confirmed the presence of two NPA motifs (Table S1). Transmembrane domain prediction tools identified six signature TM domains in 24 out of 29 AQPs (Table S2).

**Figure 1:**
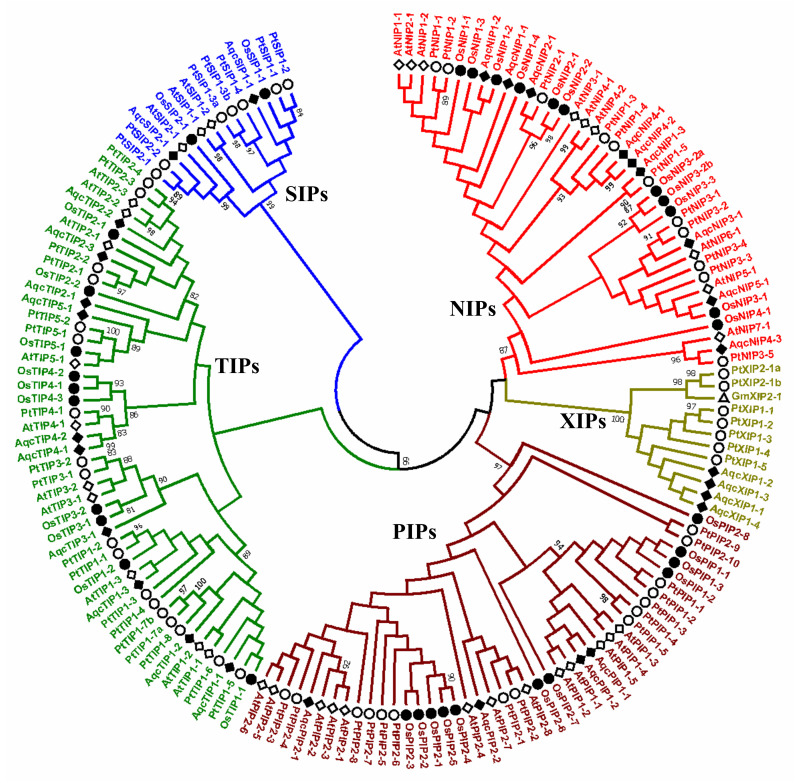
Phylogenetic analysis of aquaporins (AQPs) identified in the *Aquilegia coerulea* genome along with AQPs previously identified in rice, Arabidopsis and poplar genomes. Phylogenetic tree showing distribution of AQPs in five different subfamilies, namely, NOD26-like intrinsic proteins (NIPs; red), tonoplast intrinsic proteins (TIPs; green), plasma membrane intrinsic proteins (PIPs; maroon), small basic intrinsic proteins (SIPs; blue) and uncharacterized intrinsic proteins (XIPs; gold). The genes from Arabidopsis, rice, poplar, soybean and *Aquilegia* are indicated with the prefixes At (*Arabidopsis thaliana*, hollow square), Os (*Oryza sativa*, filled circle), Pt (*Populus trichocarpa*, hollow circle), Gm (*Glycine max*) XIPs (hollow triangle) and Aqc (*Aquilegia coerulea*, filled square), respectively.

**Figure 2:**
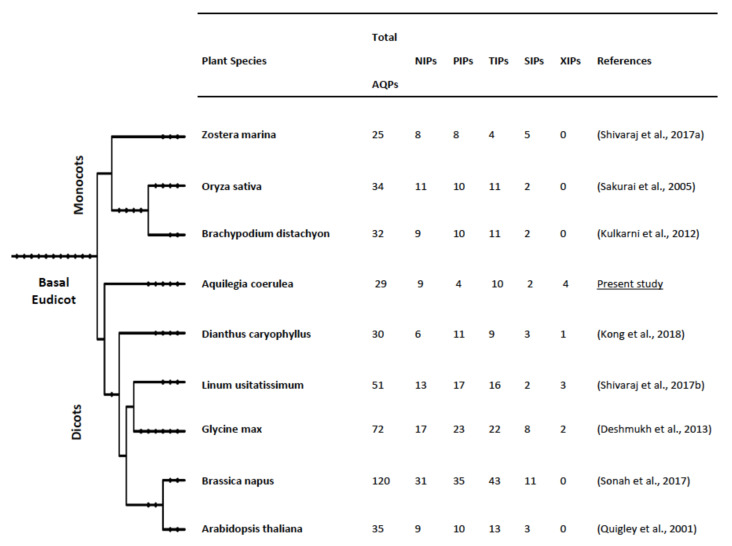
Distribution of the aquaporins and its sub-families, namely, PIPs (plasma membrane intrinsic proteins), TIPs (tonoplast intrinsic proteins), NIPs (nodulin 26-like intrinsic protein), SIPs (small basic intrinsic proteins), and XIPs (uncharacterized intrinsic proteins) in diverse plant species belonging to monocots and dicots. Phylogeny of the species was constructed using the Phylogenetic tree generator (http://phylot.biobyte.de/).

Phylogenetic analysis of *Aquilegia* AQPs using the maximum likelihood (ML) method was performed along with the known AQPs from Arabidopsis, rice, and poplar, and revealed five distinct subfamilies: PIP, TIP, NIP, SIP, and XIP (Figure 1). Based on the phylogenetic distribution and homology with known AQPs, the nomenclature of 29 *Aquilegia* AQPs was assigned (Figure 1, Figure S1). The largest subfamily, TIP, comprises ten genes that were further divided into five distinct groups (Figure S1). The nomenclature for the second-largest subfamily, NIP, was performed based on the similarity with rice or Arabidopsis NIPs. Therefore, a similar nomenclature followed for the NIPs in *Aquilegia*. The third and fourth subfamilies, PIP and SIP, both contained two groups. All the four members of the XIP family clustered together in the XIP1 group. The XIP2 group was entirely absent in *Aquilegia* when compared to the other three species used in the phylogenetic tree (Figure 1, Figure S1). Comparisons of AQPs previously identified in 25 plant species have also indicated the entire loss of one of the two XIP group in *Citrus clementina*, *Cajanus cajan*, and many others [1]. The number of XIPs was more in aquilegia compared to other species (Figure 2). The XIP family is absent in monocots and some of the dicots (Figure 2). Information about the taxonomical distribution of AQPs in different plant species, including basal eudicots, provides the basis for an evolutionary understanding of the subfamilies (Figure 2).

### 3.2. Gene Structure and Conserved Motif Analysis of Aquaporins in Aquilegia coerulea

The considerable variation of amino acid residues in the NPA motifs, ar/R SF, and Froger’s residues was observed across *Aquilegia* AQPs (Table 1). Most of the AQPs contained dual NPA motifs, with the exception of seven genes that show a variation at the first or last amino acid of the NPA motif. All the members of the PIP and TIP subfamilies possessed the conserved NPA motifs. In the NIP subfamily, seven out of nine members showed the conserved NPA motifs, but two genes of this subfamily showed a variation in one of the two NPA motifs. In the case of NIP4-3, serine was substituted for arginine in the first NPA motifs, changing it to NPS. Contrastingly, in the case of NIP5-1, valine was substituted for arginine at the second NPA motif, making it NPV (asparagine-proline-valine) instead of NPA. The presence of NIP2s with required selective filters and 108 spacing between two NPA motifs suggest its ability to uptake silicon. In the SIP subfamily, SIP1-1 showed the substitution of threonine instead of alanine (NPT), and SIP2-1 showed the substitution of leucine at the first NPA motif (NPL). In the XIP subfamily, three out of four members showed substitution of valine in the first NPA motif and substitution of threonine in the case of XIP1-4 (Table 1). Interestingly, XIP1-1 also showed substitution of histidine at the second NPA motif, making it HPA (histidine-proline-alanine) instead of NPA, which has not been reported previously.

### 3.3. Subcellular Localization and Exon–Intron Organization of Aquilegia Aquaporins

**Figure 3:**
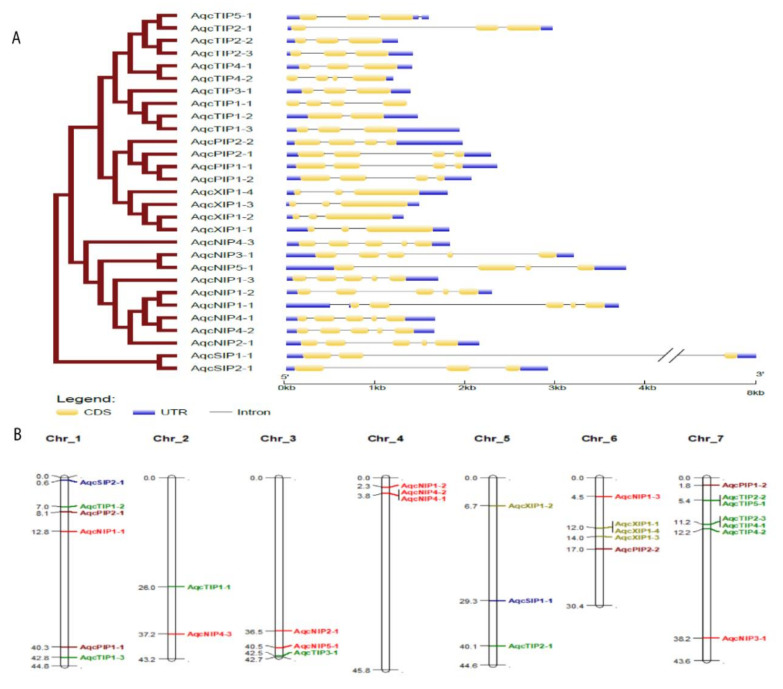
Intron–exon organization and chromosomal location of *Aquilegia* aquaporins (AQPs) (**A**). Graphic representation of the gene models of 29 AQPs identified from the *Aquilegia* genome shows the presence of a varied number of introns (2–5). Exons are shown as yellow boxes, untranslated regions (UTRs) in blue and introns are shown as black lines. The length of the exon and intron (bp) is indicated in kb in the *x*-axis. (**B**) The chromosomal location of all 29 *Aquilegia* AQPs is shown in the map. Different color codes were assigned to different classes of AQPs plasma membrane intrinsic proteins (PIP), nodulin 26-like intrinsic proteins (NIPs), tonoplast intrinsic proteins (TIPs), small intrinsic proteins (SIPs) and uncharacterized intrinsic proteins (XIPs). The AQPs from different subfamilies are denoted with different colors as PIP—purple; NIPs—red; TIPs—green; SIPs—blue; XIPs—gold.

The entire set of *Aquilegia* AQPs was predicted to be localized to the plasma membrane by the CELLO server, while the WoLF PSORT sever predicted vacuole localization for the seven AQPs (Table S2). Exon–intron structure analysis detected the presence of a varying number of introns among the AQPs, contributing to a variation in gene length (Figure 3A). A range of two to five introns per gene was observed. The distinct pattern of intron–exon organization observed among the *Aquilegia* AQP gene family correlated well with their phylogenetic distribution. Most of the phylogenetically related AQPs shared similar gene organization, suggesting the organization was present in their common ancestor before duplication. Based on the physical distribution of the loci, the highest number of AQPs were found on chromosome 7, while chromosome number 2 possessed the least number (Figure 3B). High levels of variation across the five AQP subfamilies for isoelectric point (PI) and molecular weight were observed. The highest molecular weight was observed in NIP2-1 (33.5 kDa), while TIP4-2 possessed the lowest molecular weight (24.4 kDa). Varying degrees of average PI were calculated for the various subfamilies, with NIPs having average PI of 6.42; PIPs with 8.4; and XIPs with 6.62 (Figure S2). SIPs showed the highest value for PI at 9.3, while the lowest PI of 5.9 was observed in TIPs (Table S3).

### 3.4. Characteristic Secondary and Tertiary Protein Structure of Aquilegia Aquaporins

**Figure 4:**
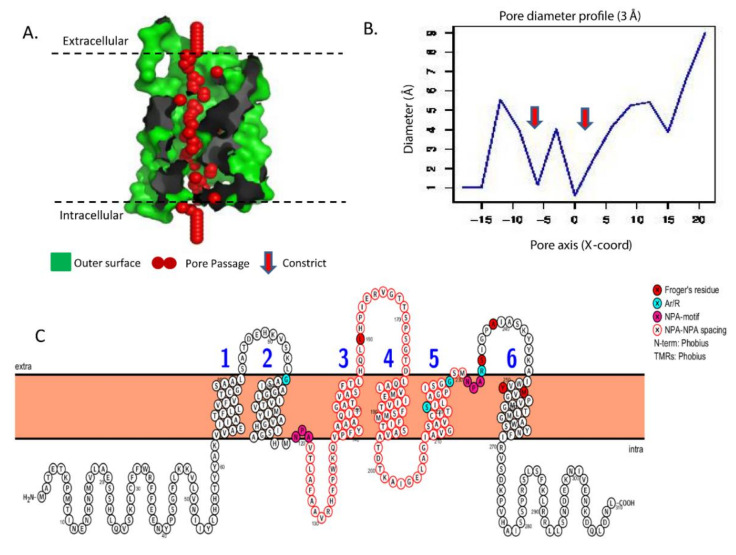
Predicted pore morphology of AqcNIP2-1 aquaporin identified in the *Aquilegia* genome. (**A**) Protein tertiary structure showing the pore morphology of AqcNIP2-1 along with features of the cavity, where red spheres represent the pore centers at a 1-Å step along the pore; (**B**) the pore diameter profile showing pore dimensions; (**C**) two-dimensional (2D) structure of AqcNIP2-1 showing an NPA motif, NPA–NPA spacing, an Ar/R selectivity filter, and Froger’s residue.

**Figure 5:**
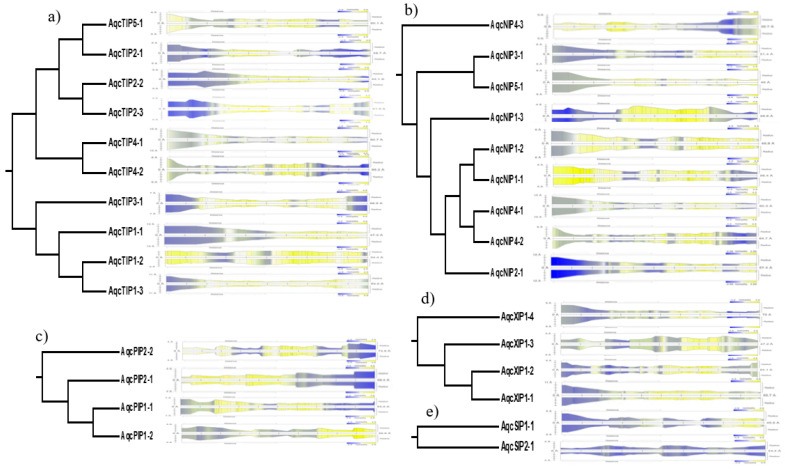
Predicted pore structure and hydropathy analysis of different subgroups: (**a**) tonoplast intrinsic proteins (TIPs); (**b**) nodulin 26-like intrinsic proteins (NIPs); (**c**) plasma membrane intrinsic proteins (PIP); (**d**) uncharacterized intrinsic proteins (XIPs); and (**e**) small intrinsic proteins (SIPs) in *Aquilegia*. In the given hydropathy plot, blue signifies the hydrophilic nature of the pore, and yellow signifies the hydrophobic nature of the pore.

The 2D structure analysis of the *Aquilegia* AQPs provided the key to understand the basic structure and organization, such as the visualization and prediction of NPA spacing, Ar/R selectivity filters, and Froger’s residue. The homology-based tertiary (3D) protein structure of all 29 *Aquilegia* AQPs was predicted to confirm 6 TM domains, hourglass-like configuration, and transcellular pores capable of transporting solutes (Figure 4A,B). The entire set of *Aquilegia* AQPs showed the presence of six TM domains, typically arranged to form an hourglass-like structure (Figure 4C). A transmembrane pore was also predicted in all the AQPs. Pore morphology studied using the MOLE2 software tool showed vast diversity for the pore-lining residues in terms of channel radius, length, polarity, charge, hydrophobicity, and hydropathy (Table 2). Based on the biochemical properties of pore-lining residues, regional hydrophobicity across the pore was predicted (Figure 5). In TIPs, hydrophilic pore openings towards both ends and a hydrophobic nature in the middle are generally uniform features. The only notable exception was TIP1-2, where the pore ends were observed to be hydrophobic, but the middle region was hydrophilic (Figure 5). Another interesting observation is the presence of hydrophilic residues at one end but a hydrophobic nature at the opposite end, for instance, in NIP1-1, NIP4-3, and PIP2-1. More interestingly, SIP2-1 has unique hydrophilic pores and lacks a hydrophobic region (Figure 5).

### 3.5. RNA-Seq Data Analysis

**Figure 6:**
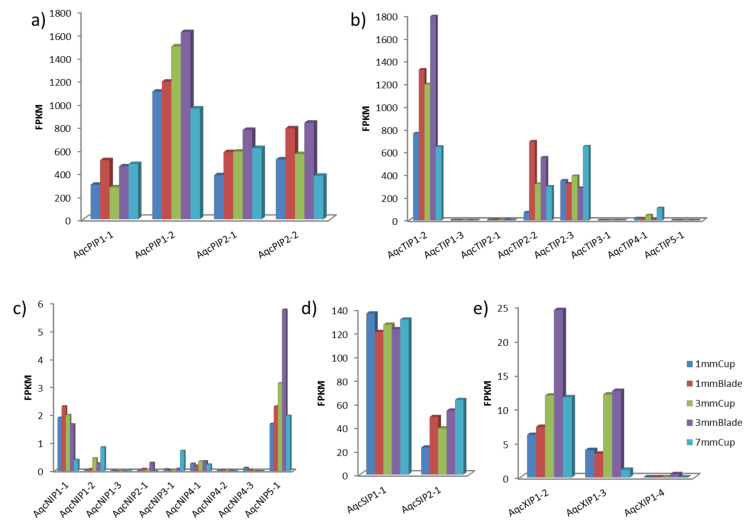
Expression of *Aquilegia* aquaporins using RNA-seq data. The normalized expression values in terms of fragments per kilobase of transcript per million mapped reads (FPKM) in (**a**) plasma membrane intrinsic proteins (PIP), (**b**), tonoplast intrinsic proteins (TIPs, (**c**) nodulin 26-like intrinsic proteins (NIPs), (**d**) small intrinsic proteins (SIPs), and (**e**) uncharacterized intrinsic proteins (XIPs)). Early developmental stages of *Aquilegia*’s petal spur were studied in a 1-mm cup, a 3-mm cup, a 7-mm cup, a 1-mm blade, and a 3-mm blade of petal spurs.

Previously published transcriptome data of three early developmental stages of the *Aquilegia* petal spur cup and blade were analyzed to understand the expression dynamics of AQPs [29]. A total of 26 out of 29 AQPs were expressed in at least one of the tissues/stages. The comparison of normalized RNA-seq data in terms of FPKM (fragment per kilobase of transcript per million mapped reads) identified the TIPs and PIPs as highly expressed AQPs subfamilies. In particular, TIP1-2 and PIP1-2 were found to be the most highly expressed AQPs (Figure 6). The magnitude of expression observed in TIPs and PIPs was several-fold higher than the other subfamilies. For instance, the most highly expressed member of TIP showed about 1792 FPKM in a 3-mm blade, PIP about 1492 FPKM in a 3-mm cup and SIP about 136 FPKM in a 1-mm cup, whereas the most highly expressed genes from NIPs and XIPs have 5.75 and 24.42 FPKM values, respectively. Since RNA expression does not necessarily correspond to protein presence and functionality, additional proteomic efforts are needed to confirm the AQP abundance.

To compare the variation of specific AQP expressions across different tissues, data visualized in the form of a heatmap were studied. In the 1-mm cup, TIP1-3, SIP1-1, and XIP1-4 showed higher expressions, whereas in the 3-mm cup TIP3-1, PIP1-2, NIP4-1, XIP1-3, and NIP4-2 showed higher expressions (Figure S3). In the 7-mm cup, NIP2-1, TIP2-3, TIP4-1, NIP3-1, NIP1-2 were highly expressed, suggesting a potential association with the developing nectary and a transition to cell elongation in the spur. In the 1-mm blade, TIP2-2, NIP1-3, NIP4-2, and XIP1-4 showed higher expression, while in the 3-mm blade, NIP5-1 and XIP1-2 were highly expressed compared to other tissues.

## 4. Discussion

In the present study, genome-wide identification and characterization of AQPs were performed in *Aquilegia* to understand the evolution and molecular role of this family, particularly in the development of the petal nectar spur. When compared to animals, plants usually possess a higher number of AQPs, which have evolved into specific subfamilies that differ in solute specificity, subcellular localization, and molecular functions [12]. Plant AQPs are also diversified in terms of their expression profiling, with specific patterns in various tissues and under certain environmental conditions [13,15]. The higher number of AQPs in plants compared to animals is probably due to the higher frequency of whole-genome duplication events during plant evolution [18], and also possibly due to the plant physiology and sessile nature of life. The AQP gene family appears to be more diverse in moss and spike moss lineages compared to the seed plants [12,49]. For instance, moss and spike moss have seven subfamilies, including two additional HIP and GIP, along with the five subfamilies of AQPs found in most of the seed plants, including *Aquilegia*. Among the seed plants, variation was observed in the distribution of five subfamilies. The entire monocot clade, as well as some dicot families such as the Brassicaceae, have lost the entire XIPs lineage [1,15,50]. The absence of the XIPs in rice and Arabidopsis, the two extensively studied model plants, limits the efforts to characterize the functions of XIPs when compared to other subfamilies. In this regard, *Aquilegia* may serve as a useful model, first, because *Aquilegia* is roughly phylogenetically equidistant from monocots and dicots and, secondly, it has only lost the XIP2s lineage. The absence of the entire XIP subfamily has been reported in several studies; however, loss of a single group from XIP has not been previously reported. The occurrence of XIP1s only in basal eudicots like *Aquilegia* suggests a subsequent loss in monocots and diversions of two distinct XIP groups in the majority of dicots. As with *Aquilegia*, three other species were found to have only one XIP, which provides an opportunity to study the XIP’s role more efficiently. The higher number of XIPs may share the functional role among the members, or they may work together in a dose-dependent manner, but having only one XIP may have a more versatile role.

In *Aquilegia*, the PIP subfamily is differentiated into two groups, as observed in all previous studies. However, the TIPs were grouped into five groups that were comparable to only one group observed in *Physcomitrella patens* [51]. The observations suggest that the diversification of PIPs occurred much earlier than that of TIPs.

Among NIPs, NIP2 (NIP-III) involved in silicon transport was identified in the *Aquilegia,* but was reported to be absent from many dicot species, including the entire Brassicaceae family. An earlier study has shown that only NIP-III present in the plant species is capable of accumulating a significant amount of silicon (>0.5%) under a condition of supplemented Si [52,53,54,55]. Furthermore, some exceptions have been observed; tomato and citrus, for example, are unable to uptake Si. The inability of these species appears to be associated with the deviation from 108 amino acid spacing between the NPA motifs [1]. Based on the NPA spacing criteria, several novel Si transporters have been identified. Interestingly, NIP-IIIs from horsetails, a primitive plant species which accumulates over 10% Si, have precise 108 amino acid spacing [56,57]. To date, no exception was observed where NIP-III with 108 NPA spacing was absent, but the plant species are able to uptake a significant amount of Si [58,59]. The presence of NIP-III (NIP2-1) with exact 108 NPA spacing indicates that *Aquilegia* species are capable of Si uptake. However, to confirm the predisposition, experimental validation is required.

Gene structure organization is helpful to illustrate the evolution of a gene family. As expected, the similar exon–intron organization was observed in the phylogenetically close homologs. This pattern of conserved exon–intron organization of AQPs has also been observed in several plant genomes, including rice, *Arabidopsis*, soybean, brassica, and flax [1,12,13,14,60,61]. Overall, two to five intron per gene is the most frequently observed structure across the species. None of the *Aquilegia* AQPs were intron-less, as observed previously. However, intron-less genes are predicted to be newly evolved compared to genes abundant in intron [62]. The intron-less genes are thought to be evolved through retrotransposon activity. All the AQPs with introns suggest a shared ancestral origin and indicate at least the role of a retrotransposon in AQP family expansion. As with exon–intron organization, biochemical and physical properties of AQPs were also well aligned with the phylogenetic distribution. Molecular weight and pI [63] of AQPs are indicative of their subcellular localization. Recently, proteomic analysis in the Arabidopsis genome revealed a relatively lower pI (6.69) for vacuolar proteins when compared to all other proteins (pI 7.40). This is consistent with the low PI for *Aquilegia* TIPs compared to PIPs and SIPs, as observed in the present study.

Usually, the solute specificity of the AQPs is determined by the pore-lining amino acids and constriction. Solute specificity of the AQPs has largely been predicted based on conserved amino acids present at ar/R SF, NPA domains, and Forger’s residues (Figure 4). These conserved positions are usually predicted based on sequence alignment. However, protein 3D structure has never been considered for the solute prediction. In this regard, the pore morphology studied in the present study will provide novel insight for the prediction of solute specificity. The pore morphology and hydropathy plots showed varying degrees of hydrophobic and hydrophilic regions in the AQPs pertaining to the transport of hydrophilic or hydrophobic solutes. The physicochemical analysis of the pore showed a high variation in polarity and hydropathy, which signifies the specificity of the various AQPs to different solutes (Table 2).

The RNA-seq analysis provided insights into the expression patterns of the various AQPs during different stages of early spur development. Recently, it has been reported that the spur development in *Aquilegia* is the result of localized cell division at the early stages, followed by prolonged anisotropic cell elongation [29,64]. In the present study, higher expression of TIPs and PIPs in the developing spur cup relative to the blade signifies a potential role of AQPs during these developmental stages of the spur. The PIPs and TIPs are known to be highly permeable to water [65]. Hence, their abundance indicated their role in the spur. In *Aquilegia*, TIPs from different groups were found highly expressed in cups at a different stage of development, indicating a role in spur development, consistent with previous studies showing that TIPs are highly involved in the cell division and elongation process. In Arabidopsis, the expression of the β-glucuronidase (GUS) protein under the AtTIP1;1 promoter showed high activity in stem- and root-elongating tissues [66]. The tightly regulated expression of AtTIP1;2 and AtTIP2;1, leading to spatial and temporal control of the cellular water transport, which is essential during the highly regulated lateral root primordium morphogenesis and emergence. Increased expression of TIPs is also observed in elongating hypocotyls in different crop plants [31,67,68]. In *Zea mays*, abundant expression of ZmTIP1;1 is observed in expanding cells in roots, leaves, and reproductive organs [69,70]. In a 3-mm cup of *Aquilegia*, *PIP1-2* showed higher expression, similar to the higher expression of DcaPIP1;1 DcaPIP1;3 previously observed in *Dianthus* during the flower opening stages [71]. In tulips [72,73] and roses [74], members of the PIP1 subfamily have been shown to be involved in water transport and petal expansion.

The XIPs are known to transport several solutes such as glycerol and boric acid in the plants. The characterization of abundantly expressing XIPs with respect to solute permeability is important to establish their role in spur elongation. Notably, the role of boron in flower development is well established in Arabidopsis and Brassica species [75]. The hypothesis for a probable role of XIPs in the development of petal nectar spurs in *Aquilegia* needs further investigation.

## 5. Conclusions

The genome-wide analysis and characterization of AQPs in *Aquilegia* will be helpful in understanding the evolution of the gene family across angiosperms. The occurrence of only one group in the XIP subfamily suggests the possibility of an independent loss of the entire XIP family from monocots and some of the dicot families (Brassicacea, for example), and further expansion into two groups in the majority of dicots. The expression pattern of AQPs evaluated during different stages of early spur development was helpful in illustrating the probable role. The higher expression of PIPs and TIPs observed in the developing spur indicates the role in cell elongation and differentiation, as previously observed in the root development. Additional efforts are needed to confirm the expression profiling performed here using available RNAseq data. Furthermore, extensive analysis of AQPs revealed the variation in the pore radius, morphology, and hydropathy, which will be suggestive for the prediction of the solute specificity. The AQPs identified in the present study provide an ample amount of information for further characterization and investigation to understand their role in the various developmental stages and physiological processes.

## Figures and Tables

**Table 1:** Details of conserved domains, selectivity filter, and amino acid residues of aquaporins identified in the *Aquilegia* genome.

Gene ID	NPA Motifs		NPA Spacing	Ar/R Selectivity Filters				Froger’s Residues					Mitani’s Residue
	NPA (LB)	NPA (LE)		H2	H5	LE1	LE2	P1	P2	P3	P4	P5	
**AqcNIP1-1**	NPA	NPA	109	W	V	A	R	F	S	A	Y	I	P
**AqcNIP1-2**	NPA	NPA	109	W	V	A	R	F	S	A	Y	I	P
**AqcNIP1-3**	NPA	NPA	112	W	V	S	R	Y	S	A	Y	L	P
**AqcNIP2-1**	NPA	NPA	108	G	S	G	R	L	S	A	Y	M	P
**AqcNIP3-1**	NPA	NPA	108	T	I	A	R	F	S	A	Y	L	P
**AqcNIP4-1**	NPA	NPA	109	W	V	A	R	F	S	A	Y	M	P
**AqcNIP4-3**	NPS	NPA	108	G	I	G	R	Y	S	A	Y	I	P
**AqcNIP4-2**	NPA	NPA	109	W	V	A	R	F	S	A	Y	I	P
**AqcNIP5-1**	NPS	NPV	108	A	I	G	R	F	T	A	Y	L	P
**AqcPIP1-1**	NPA	NPA	118	F	H	T	R	Q	S	A	H	W	W
**AqcPIP1-2**	NPA	NPA	118	F	H	T	R	Q	S	A	H	W	W
**AqcPIP2-1**	NPA	NPA	118	F	H	T	R	Q	S	A	Q	W	W
**AqcPIP2-2**	NPA	NPA	118	F	H	T	R	M	S	A	H	W	W
**AqcSIP1-1**	NPT	NPA	22	V	G	L	A	Q	I	G	E	Y	Q
**AqcSIP2-1**	NPL	NPA	40	S	A	L	K	L	Q	V	E	E	E
**AqcTIP1-2**	NPA	NPA	111	H	I	A	V	T	A	A	Y	W	P
**AqcTIP1-1**	NPA	NPA	130	A	I	A	V	T	S	A	Y	W	P
**AqcTIP1-3**	NPA	NPA	111	H	I	A	V	T	S	A	Y	W	P
**AqcTIP2-1**	NPA	NPA	110	H	I	G	R	T	S	A	Y	W	P
**AqcTIP2-3**	NPA	NPA	111	H	I	G	R	T	S	A	Y	W	P
**AqcTIP2-2**	NPA	NPA	111	H	I	G	R	T	S	A	Y	W	P
**AqcTIP3-1**	NPA	NPA	111	H	I	A	R	T	A	A	Y	W	P
**AqcTIP4-1**	NPA	NPA	111	H	I	A	R	T	S	S	Y	W	P
**AqcTIP4-2**	NPA	NPA	96	H	I	A	R	S	S	S	Y	W	P
**AqcTIP5-1**	NPA	NPA	110	S	V	G	C	T	S	A	Y	W	P
**AqcXIP1-2**	NPV	NPA	135	I	V	V	R	V	C	A	W	V	V
**AqcXIP1-1**	NPV	HPA	135	V	V	V	R	I	C	A	W	I	V
**AqcXIP1-4**	NPT	NPA	134	V	V	V	R	V	C	T	W	I	V
**AqcXIP1-3**	NPA	NPA	135	I	V	V	R	V	C	A	W	V	V

LB—loop B; LE—loop E; H2 and H5—transmembrane helix; P1–5—positions; NPA- asparagine-proline-alanine; NPV- asparagine-proline-valine; NPT- asparagine-proline-threonine; NPL- asparagine-proline-leucine; NPS- asparagine-proline-serine; HPA- histidine-proline-alanine.

**Table 2:** Physicochemical properties of the pore structure predicted for the *Aquilegia* aquaporins (AQPs). Three-dimensional structures of AQPs were predicted by using the Phyre2 server and subsequently visualized and analyzed using the Mole 2.5 server to predict the physicochemical properties of AQP pores, such as hydropathy, bottleneck, polarity, and mutability.

Aquaporins	Length(Å)	Hydropathy	Bottleneck	Charge	Polarity	Mutability	LogP	LogD	LogS	Ionizable
AqcNIP1-1	56.40	0.63	0.80	1.00	9.26	91.00	0.61	0.35	−0.19	3.00
AqcNIP1-2	49.80	0.98	1.00	2.00	7.05	84.00	0.74	0.54	−0.39	4.00
AqcNIP1-3	38.80	1.08	0.30	1.00	6.10	91.00	0.65	0.43	−0.22	3.00
AqcNIP2-1	57.40	−0.03	1.20	2.00	9.50	91.00	0.27	0.00	0.32	4.00
AqcNIP3-1	51.40	0.08	1.30	1.00	6.02	91.00	0.26	0.06	0.03	1.00
AqcNIP4-1	57.60	0.31	0.60	−1.00	10.39	87.00	0.47	0.12	0.02	5.00
AqcNIP4-3	38.70	0.37	−0.10	−2.00	8.08	90.00	0.17	−0.16	0.34	2.00
AqcNIP4-2	84.70	0.16	0.70	−1.00	6.68	91.00	0.30	0.10	0.15	3.00
AqcNIP5-1	46.00	0.98	0.20	−1.00	4.22	86.00	0.59	0.46	−0.03	1.00
AqcPIP1-1	83.20	0.38	1.10	0.00	11.40	82.00	0.70	0.30	−0.17	8.00
AqcPIP1-2	35.50	0.39	0.20	−1.00	5.21	81.00	0.44	0.39	−0.12	1.00
AqcPIP2-1	38.40	0.14	0.60	1.00	8.44	83.00	0.90	0.71	−0.46	3.00
AqcPIP2-2	74.90	0.22	0.00	1.00	8.60	89.00	0.66	0.34	−0.14	3.00
AqcSIP1-1	45.50	−0.85	0.00	−1.00	3.89	81.00	0.30	0.28	−0.09	1.00
AqcSIP2-1	43.40	−1.83	0.40	8.00	18.48	85.00	N/A	−0.73	0.62	8.00
AqcTIP1-2	34.40	1.09	1.20	2.00	7.19	82.00	0.93	0.79	−0.64	2.00
AqcTIP1-1	47.30	1.21	1.00	0.00	4.79	92.00	0.61	0.55	−0.28	2.00
AqcTIP1-3	53.20	1.11	0.50	1.00	6.09	85.00	0.58	0.50	−0.23	3.00
AqcTIP2-1	48.70	−0.10	0.00	1.00	10.98	85.00	0.32	−0.02	0.07	3.00
AqcTIP2-3	61.80	0.54	0.50	2.00	8.66	86.00	0.49	0.34	−0.21	2.00
AqcTIP2-2	44.10	0.89	0.50	2.00	8.70	84.00	0.58	0.39	−0.29	2.00
AqcTIP3-1	58.50	0.81	0.50	3.00	8.74	86.00	0.62	0.42	−0.25	5.00
AqcTIP4-1	60.70	0.36	0.60	−1.00	9.79	87.00	0.47	0.14	−0.01	5.00
AqcTIP4-2	98.20	−0.04	0.70	−2.00	8.54	90.00	0.33	0.04	0.20	6.00
AqcTIP5-1	60.10	0.67	0.60	1.00	4.05	84.00	0.32	0.28	−0.09	1.00
AqcXIP1-2	94.10	0.24	1.00	4.00	10.58	84.00	0.59	0.12	−0.04	6.00
AqcXIP1-1	55.70	0.60	0.70	1.00	9.16	86.00	0.81	0.60	−0.35	3.00
AqcXIP1-4	70.00	−0.31	0.60	4.00	10.05	86.00	0.44	0.03	0.04	4.00
AqcXIP1-3	47.20	0.00	0.20	1.00	7.66	89.00	0.23	0.06	0.12	1.00

## Supplementary Materials

The following are available online at https://www.mdpi.com/2223-7747/9/6/799/s1, Figure S1: Phylogenetic analysis of *Aquilegia coerulea* aquaporins (AQPs). Phylogenetic tree showing the distribution of aquaporins in five different subfamilies namely NOD26-like intrinsic proteins (NIPs), tonoplast intrinsic proteins (TIPs), plasma membrane intrinsic proteins (PIPs), small basic intrinsic proteins (SIPs) and uncharacterized intrinsic proteins (XIPs). The outgroup was taken from *Glycine max* (Gm)., Figure S2: Molecular weight and isoelectric point of *Aquilegia coerulea* aquaporins (AQPs)., Figure S3: Heatmap showing expression profile of aquaporin genes in petal spur of AquilIegia coIerula. Genes with no expression (0 FPKM values) were removed from the heatmap. Table S1: Conserved domain analysis of AQPs identified from *Aquilegia coerula* using CDD tool from NCBI. Table S2: Transmembrane domains and cellular localization of AQPs identified from *Aquilegia coerula* using TMHMM, TargetP, Cello and wolfpsort. Table S3: Molecular weight and average Isoelectric point (PI) of *Aquilegia coerula* Aquaporins.

## References

[1] Deshmukh R.K., Vivancos J., Ramakrishnan G., Guérin V., Carpentier G., Sonah H., Labbé C., Isenring P., Belzile F.J., Bélanger R.R. (2015). A precise spacing between the NPA domains of aquaporins is essential for silicon permeability in plants. Plant J..

[2] Hove R.M., Bhave M. (2011). Plant aquaporins with non-aqua functions: Deciphering the signature sequences. Plant Mol. Biol..

[3] Maurel C., Javot H., Lauvergeat V., Gerbeau P., Tournaire C., Santoni V., Heyes J. (2002). Molecular physiology of aquaporins in plants. International Review of Cytology.

[4] Deshmukh R.K., Nguyen H.T., Belanger R.R. (2017). Editorial: Aquaporins: Dynamic Role and Regulation. Front. Plant Sci..

[5] Laloux T., Junqueira B., Maistriaux L.C., Ahmed J., Jurkiewicz A., Chaumont F. (2018). Plant and Mammal Aquaporins: Same but Different. Int. J. Mol. Sci..

[6] Preston G.M., Agre P. (1991). Isolation of the cDNA for erythrocyte integral membrane protein of 28 kilodaltons: Member of an ancient channel family. Proc. Natl. Acad. Sci. USA.

[7] Preston G.M., Carroll T.P., Guggino W.B., Agre P. (1992). Appearance of water channels in Xenopus oocytes expressing red cell CHIP28 protein. Science.

[8] Papadopoulos M.C., Verkman A.S. (2013). Aquaporin water channels in the nervous system. Nat. Rev. Neurosci..

[9] Benga O., Huber V.J. (2012). Brain water channel proteins in health and disease. Mol. Asp. Med..

[10] King L.S., Kozono D., Agre P. (2004). From structure to disease: The evolving tale of aquaporin biology. Nat. Rev. Mol. Cell Biol..

[11] Singh R.K., Deshmukh R., Muthamilarasan M., Rani R., Prasad M. (2020). Versatile roles of aquaporin in physiological processes and stress tolerance in plants. Plant Physiol. Biochem..

[12] Deshmukh R.K., Sonah H., Bélanger R.R. (2016). Plant Aquaporins: Genome-wide identification, transcriptomics, proteomics, and advanced analytical tools. Front. Plant Sci..

[13] Sonah H., Deshmukh R.K., Labbé C., Bélanger R.R. (2017). Analysis of aquaporins in Brassicaceae species reveals high-level of conservation and dynamic role against biotic and abiotic stress in canola. Sci. Rep..

[14] Quigley F., Rosenberg J.M., Shachar-Hill Y., Bohnert H.J. (2001). From genome to function: The Arabidopsis aquaporins. Genome Biol..

[15] Vishwakarma K., Mishra M., Patil G., Mulkey S., Ramawat N., Singh V P., Deshmukh R., Kumar Tripathi D.K., Nguyen H.T., Sharma S. (2019). Avenues of the membrane transport system in adaptation of plants to abiotic stresses. Crit. Rev. Biotech..

[16] Guan X.G., Su W.H., Yi F., Zhang D., Hao F., Zhang H.G., Liu Y.J., Feng X.C., Ma T.H. (2010). NPA motifs play a key role in plasma membrane targeting of aquaporin-4. IUBMB Life.

[17] Santoni V., Joëlle V., Pflieger D., Sommerer N., Maurel C. (2003). A proteomic study reveals novel insights into the diversity of aquaporin forms expressed in the plasma membrane of plant roots. Biochem. J..

[18] Shivaraj S., Deshmukh R., Sonah H., Bélanger R.R. (2019). Identification and characterization of aquaporin genes in Arachis duranensis and Arachis ipaensis genomes, the diploid progenitors of peanut. BMC Genom..

[19] Maurel C., Boursiac Y., Luu D.-T., Santoni V., Shahzad Z., Verdoucq L. (2015). Aquaporins in plants. Physiol. Rev..

[20] Vera-Estrella R., Barkla B.J., Amezcua-Romero J.C., Pantoja O. (2012). Day/night regulation of aquaporins during the CAM cycle in Mesembryanthemum crystallinum. Plant Cell Environ..

[21] Törnroth-Horsefield S., Wang Y., Hedfalk K., Johanson U., Karlsson M., Tajkhorshid E., Neutze R., Kjellbom P. (2006). Structural mechanism of plant aquaporin gating. Nature.

[22] Eriksson U.K., Fischer G., Friemann R., Enkavi G., Tajkhorshid E., Neutze R. (2013). Subangstrom resolution X-ray structure details aquaporin-water interactions. Science.

[23] Kramer E.M. (2009). Aquilegia: A new model for plant development, ecology, and evolution. Annu. Rev. Plant Biol..

[24] Filiault D.L., Ballerini E.S., Mandáková T., Aköz G., Derieg N.J., Schmutz J., Jenkins J., Grimwood J., Shu S., Hayes R.D. (2018). The Aquilegia genome provides insight into adaptive radiation and reveals an extraordinarily polymorphic chromosome with a unique history. eLife.

[25] Brunet J., Eckert C. (1998). Effects of floral morphology and display on outcrossing in blue columbine, Aquilegia caerulea (Ranunculaceae). Funct. Ecol..

[26] Miller R.B. (1981). Hawkmoths and the geographic patterns of floral variation in Aquilegia caerulea. Evolution.

[27] Sharma B., Kramer E.M. (2014). The MADS-box gene family of the basal eudicot and hybrid *Aquilegia coerulea* ‘Origami’ (Ranunculaceae). Ann. Mo. Bot. Gard..

[28] Thairu M.W., Brunet J. (2015). The role of pollinators in maintaining variation in flower colour in the Rocky Mountain columbine, Aquilegia coerulea. Ann. Bot..

[29] Yant L., Collani S., Puzey J., Levy C., Kramer E.M. (2015). Molecular basis for three-dimensional elaboration of the Aquilegia petal spur. Proc. R. Soc. Lond. B Biol. Sci..

[30] Higuchi T., Suga S., Tsuchiya T., Hisada H., Morishima S., Okada Y., Maeshima M. (1998). Molecular cloning, water channel activity and tissue specific expression of two isoforms of radish vacuolar aquaporin1. Plant Cell Physiol..

[31] Ishibashi K., Kondo S., Hara S., Morishita Y. (2010). The evolutionary aspects of aquaporin family. Am. J. Physiol. Regul. Integr. Comp. Physiol..

[32] Siefritz F., Otto B., Bienert G.P., Van Der Krol A., Kaldenhoff R. (2004). The plasma membrane aquaporin NtAQP1 is a key component of the leaf unfolding mechanism in tobacco. Plant J..

[33] Martre P., Morillon R., Barrieu F., North G.B., Nobel P.S., Chrispeels M.J. (2002). Plasma membrane aquaporins play a significant role during recovery from water deficit. Plant Physiol..

[34] Gupta A.B., Sankararamakrishnan R. (2009). Genome-wide analysis of major intrinsic proteins in the tree plant Populus trichocarpa: Characterization of XIP subfamily of aquaporins from evolutionary perspective. BMC Plant Biol..

[35] Goodstein D.M., Shu S., Howson R., Neupane R., Hayes R.D., Fazo J., Mitros T., Dirks W., Hellsten U., Putnam N. (2011). Phytozome: A comparative platform for green plant genomics. Nucleic Acids Res..

[36] Hall T.A. (1999). BioEdit: A user-friendly biological sequence alignment editor and analysis program for Windows 95/98/NT. Nucleic Acids Symposium Series.

[37] Kumar S., Stecher G., Tamura K. (2016). MEGA7: Molecular evolutionary genetics analysis version 7.0 for bigger datasets. Mol. Biol. Evol..

[38] Bernsel A., Viklund H., Hennerdal A., Elofsson A. (2009). TOPCONS: Consensus prediction of membrane protein topology. Nucleic Acids Res..

[39] Bailey T.L., Williams N., Misleh C., Li W.W. (2006). MEME: Discovering and analyzing DNA and protein sequence motifs. Nucleic Acids Res..

[40] Emanuelsson O., Nielsen H., Brunak S., Von Heijne G. (2000). Predicting subcellular localization of proteins based on their N-terminal amino acid sequence. J. Mol. Biol..

[41] Horton P., Park K.-J., Obayashi T., Fujita N., Harada H., Adams-Collier C., Nakai K. (2007). WoLF PSORT: Protein localization predictor. Nucleic Acids Res..

[42] Yu C.S., Lin C.J., Hwang J.K. (2004). Predicting subcellular localization of proteins for Gram-negative bacteria by support vector machines based on n-peptide compositions. Protein Sci..

[43] Guo A., Zhu Q., Chen X., Luo J.C. (2007). GSDS: A gene structure display server. Yi Chuan= Hered..

[44] Voorrips R.E. (2002). MapChart: Software for the graphical presentation of linkage maps and QTLs. J. Hered..

[45] Omasits U., Ahrens C.H., Müller S., Wollscheid B. (2013). Protter: Interactive protein feature visualization and integration with experimental proteomic data. Bioinformatics.

[46] Kelley L.A., Mezulis S., Yates C.M., Wass M.N., Sternberg M.J.E. (2015). The Phyre2 web portal for protein modeling, prediction and analysis. Nat. Protoc..

[47] Berka K., Hanák O., Sehnal D., Banáš P., Navratilova V., Jaiswal D., Ionescu C.-M., Svobodová Vařeková R., Koča J., Otyepka M. (2012). MOLE online 2.0: Interactive web-based analysis of biomacromolecular channels. Nucleic Acids Res..

[48] Pellegrini-Calace M., Maiwald T., Thornton J.M. (2009). PoreWalker: A novel tool for the identification and characterization of channels in transmembrane proteins from their three-dimensional structure. PLoS Comput. Biol..

[49] Patil G., Valliyodan B., Deshmukh R., Prince S., Nicander B., Zhao M., Sonah H., Song L., Lin L., Chaudhary J. (2015). Soybean (*Glycine max*) SWEET gene family: Insights through comparative genomics, transcriptome profiling and whole genome re-sequence analysis. BMC Genom..

[50] Shivaraj S.M., Deshmukh R., Bhat J.A., Sonah H., Bélanger R.R. (2017). Understanding Aquaporin Transport System in Eelgrass (*Zostera marina* L.), an Aquatic Plant Species. Front. Plant Sci..

[51] Danielson J.Å., Johanson U. (2008). Unexpected complexity of the aquaporin gene family in the moss Physcomitrella patens. BMC Plant Biol..

[52] Guerriero G., Deshmukh R., Sonah H., Sergeant K., Hausman J.-F., Lentzen E., Valle N., Siddiqui K.S., Exley C. (2019). Identification of the aquaporin gene family in Cannabis sativa and evidence for the accumulation of silicon in its tissues. Plant Sci..

[53] Bhat J.A., Shivaraj S., Singh P., Navadagi D.B., Tripathi D.K., Dash P.K., Solanke A.U., Sonah H., Deshmukh R. (2019). Role of silicon in mitigation of heavy metal stresses in crop plants. Plants.

[54] Deshmukh R.K., Ma J.F., Bélanger R.R. (2017). Role of silicon in plants. Front. Plant Sci..

[55] Majeed Zargar S., Nazir M., Kumar Agrawal G., Kim D.-W., Rakwal R. (2010). Silicon in plant tolerance against environmental stressors: Towards crop improvement using omics approaches. Curr. Proteom..

[56] Vivancos J., Deshmukh R., Grégoire C., Rémus-Borel W., Belzile F., Bélanger R.R. (2016). Identification and characterization of silicon efflux transporters in horsetail (*Equisetum arvense*). J. Plant Physiol..

[57] Grégoire C., Rémus-Borel W., Vivancos J., Labbé C., Belzile F., Bélanger R.R. (2012). Discovery of a multigene family of aquaporin silicon transporters in the primitive plant Equisetum arvense. Plant J..

[58] Coskun D., Deshmukh R., Sonah H., Menzies J.G., Reynolds O., Ma J.F., Kronzucker H.J., Bélanger R.R. (2018). The controversies of silicon’s role in plant biology. New Phytol..

[59] Deshmukh R., Bélanger R.R. (2016). Molecular evolution of aquaporins and silicon influx in plants. Funct. Ecol..

[60] Shivaraj S., Deshmukh R.K., Rai R., Bélanger R., Agrawal P.K., Dash P.K. (2017). Genome-wide identification, characterization, and expression profile of aquaporin gene family in flax (*Linum usitatissimum*). Sci. Rep..

[61] Zargar S.M., Nagar P., Deshmukh R., Nazir M., Wani A.A., Masoodi K.Z., Agrawal G.K., Rakwal R. (2017). Aquaporins as potential drought tolerance inducing proteins: Towards instigating stress tolerance. J. Proteom..

[62] Deshmukh R.K., Sonah H., Singh N.K. (2016). Intron gain, a dominant evolutionary process supporting high levels of gene expression in rice. J. Plant Biochem. Biotechnol..

[63] Kozlowski L.P. (2016). IPC–isoelectric point calculator. Biol. Direct.

[64] Puzey J.R., Gerbode S.J., Hodges S.A., Kramer E.M., Mahadevan L. (2012). Evolution of spur-length diversity in Aquilegia petals is achieved solely through cell-shape anisotropy. Proc. R. Soc. Lond. B Biol. Sci..

[65] Uehlein N., Fileschi K., Eckert M., Bienert G.P., Bertl A., Kaldenhoff R. (2007). Arbuscular mycorrhizal symbiosis and plant aquaporin expression. Phytochemistry.

[66] Ludevid D., Höfte H., Himelblau E., Chrispeels M.J. (1992). The expression pattern of the tonoplast intrinsic protein γ-TIP in Arabidopsis thaliana is correlated with cell enlargement. Plant Physiol..

[67] Suga S., Maeshima M. (2004). Water channel activity of radish plasma membrane aquaporins heterologously expressed in yeast and their modification by site-directed mutagenesis. Plant Cell Physiol..

[68] Eisenbarth D.A., Weig A.R. (2005). Dynamics of aquaporins and water relations during hypocotyl elongation in Ricinus communis L. seedlings. J. Exp. Bot..

[69] Barrieu F., Chaumont F., Chrispeels M.J. (1998). High Expression of the Tonoplast Aquaporin ZmTIP1in Epidermal and Conducting Tissues of Maize. Plant Physiol..

[70] Chaumont F., Barrieu F., Herman E.M., Chrispeels M.J. (1998). Characterization of a maize tonoplast aquaporin expressed in zones of cell division and elongation. Plant Physiol..

[71] Kong W., Bendahmane M., Fu X. (2018). Genome-wide identification and characterization of aquaporins and their role in the flower opening processes in carnation (*Dianthus caryophyllus*). Molecules.

[72] Azad A.K., Sawa Y., Ishikawa T., Shibata H. (2004). Phosphorylation of plasma membrane aquaporin regulates temperature-dependent opening of tulip petals. Plant Cell Physiol..

[73] Azad A.K., Katsuhara M., Sawa Y., Ishikawa T., Shibata H. (2008). Characterization of four plasma membrane aquaporins in tulip petals: A putative homolog is regulated by phosphorylation. Plant Cell Physiol..

[74] Ma N., Xue J., Li Y., Liu X., Dai F., Jia W., Luo Y., Gao J. (2008). Rh-PIP2; 1, a rose aquaporin gene, is involved in ethylene-regulated petal expansion. Plant Physiol..

[75] Zhang Q., Chen H., He M., Zhao Z., Cai H., Ding G., Shi L., Xu F. (2017). The boron transporter BnaC4. BOR1; 1c is critical for inflorescence development and fertility under boron limitation in Brassica napus. Plant Cell Environ..

