# Supplementary material for: Evolutionary Understanding of Aquaporin Transport System in the Basal Eudicot Model Species Aquilegia coerulea
Source: Plants (Basel). 2020 Jun 26;9(6):799. doi: 10.3390/plants9060799 (PMC7355465; doi:10.3390/plants9060799)
Supplement: Supplementary file 1 [file plants-09-00799-s001.pdf]

Table S1. Conserved domain analysis of AQPs identified from *Aquilegia coerula* using CDD tool from NCBI.

Query	Hit type	PSSM-ID	E-Value	Bitscore	Accession	Short name	Superfamily
**AqcNIP1-1**	superfamily	294134	6.35E-125	357.024	cl00200	MIP superfamily	-
**AqcNIP1-2**	superfamily	294134	4.39E-134	380.906	cl00200	MIP superfamily	-
**AqcNIP1-3**	superfamily	294134	1.74E-77	236.842	cl00200	MIP superfamily	-
**AqcNIP2-1**	superfamily	294134	1.20E-76	235.574	cl00200	MIP superfamily	-
**AqcNIP3-1**	Specific	177663	1.17E-161	452.009	PLN00026	PLN00026	cl00200
**AqcNIP4-1**	superfamily	294134	1.78E-132	375.786	cl00200	MIP superfamily	-
**AqcNIP4-3**	superfamily	294134	2.49E-118	340.296	cl00200	MIP superfamily	-
**AqcNIP4-2**	superfamily	294134	6.03E-132	374.631	cl00200	MIP superfamily	-
**AqcNIP5-1**	Specific	177663	3.17E-171	475.891	PLN00026	PLN00026	cl00200
**AqcPIP1-1**	Specific	278651	8.12E-99	288.832	pfam00230	MIP	cl00200
**AqcPIP1-2**	Specific	278651	5.09E-98	286.906	pfam00230	MIP	cl00200
**AqcPIP2-1**	Specific	278651	1.66E-94	277.661	pfam00230	MIP	cl00200
**AqcPIP2-2**	Specific	278651	1.82E-92	272.654	pfam00230	MIP	cl00200
**AqcSIP1-1**	superfamily	294134	5.30E-17	76.9086	cl00200	MIP superfamily	-
**AqcSIP2-1**	superfamily	294134	3.51E-08	52.2558	cl00200	MIP superfamily	-
**AqcTIP1-2**	Specific	177664	2.25E-146	409.176	PLN00027	PLN00027	cl00200
**AqcTIP1-1**	Specific	177664	6.59E-119	339.455	PLN00027	PLN00027	cl00200
**AqcTIP1-3**	Specific	177664	4.11E-155	431.132	PLN00027	PLN00027	cl00200
**AqcTIP2-1**	superfamily	294134	9.56E-79	237.913	cl00200	MIP superfamily	-
**AqcTIP2-3**	superfamily	294134	1.10E-120	343.843	cl00200	MIP superfamily	-
**AqcTIP2-2**	Specific	165733	6.48E-134	377.355	PLN00166	PLN00166	cl00200
**AqcTIP3-1**	Specific	177664	3.93E-109	314.802	PLN00027	PLN00027	cl00200
**AqcTIP4-1**	superfamily	294134	2.51E-99	289.764	cl00200	MIP superfamily	-
**AqcTIP4-2**	superfamily	294134	5.96E-82	245.081	cl00200	MIP superfamily	-
**AqcTIP5-1**	superfamily	294134	4.44E-121	346.12	cl00200	MIP superfamily	-
**AqcXIP1-2**	superfamily	294134	2.31E-36	130.066	cl00200	MIP superfamily	-
**AqcXIP1-1**	superfamily	294134	3.58E-35	126.599	cl00200	MIP superfamily	-
**AqcXIP1-4**	superfamily	294134	7.22E-40	138.926	cl00200	MIP superfamily	-
**AqcXIP1-3**	superfamily	294134	4.38E-37	131.607	cl00200	MIP superfamily	-

Table S2. Transmembrane domains and cellular localization of AQPs identified from *Aquilegia coerula* using TMHMM, Targetp, Cello and wolfpsort.

S. No.	Gene ID	Length	TMHMM			Targetp	Cello	Wolfpsort
			**ExpAA**	**First60**	**TMH**			
1	AqcNIP1-1	268	130.8	22	6		PlasmaMembrane	PlasmaMembrane
2	AqcNIP1-2	285	129.87	9.3	6		PlasmaMembrane	Vacoule
3	AqcNIP1-3	277	131.03	7.35	6		PlasmaMembrane	PlasmaMembrane
4	AqcNIP2-1	311	132.41	0.19	6		PlasmaMembrane	Vacoule
5	AqcNIP3-1	307	134.45	0.49	6	Chloroplast	PlasmaMembrane	PlasmaMembrane
6	AqcNIP4-1	271	130.66	19.42	6		PlasmaMembrane	PlasmaMembrane
7	AqcNIP4-3	289	129.36	0.05	6		PlasmaMembrane	PlasmaMembrane
8	AqcNIP4-2	278	130.96	14.79	6		PlasmaMembrane	PlasmaMembrane
9	AqcNIP5-1	301	122.52	0.05	6	Chloroplast	PlasmaMembrane	PlasmaMembrane
10	AqcPIP1-1	288	122.45	7.04	6		PlasmaMembrane	PlasmaMembrane
11	AqcPIP1-2	288	120.84	6.36	6		PlasmaMembrane	PlasmaMembrane
12	AqcPIP2-1	284	132.08	21.22	6		PlasmaMembrane	PlasmaMembrane
13	AqcPIP2-2	281	135.72	22.12	6		PlasmaMembrane	PlasmaMembrane
14	AqcSIP1-1	245	130.48	35.98	6		PlasmaMembrane	Vacoule
15	AqcSIP2-1	251	106.1	26.79	5	Mitochondria	PlasmaMembrane	PlasmaMembrane
16	AqcTIP1-2	254	148.75	23.33	7		PlasmaMembrane	PlasmaMembrane
17	AqcTIP1-1	250	128.06	19.87	6		PlasmaMembrane	PlasmaMembrane
18	AqcTIP1-3	253	135.67	24.63	6		PlasmaMembrane	Vacoule
19	AqcTIP2-1	264	143.89	22.04	6		PlasmaMembrane	PlasmaMembrane
20	AqcTIP2-3	249	153.83	29.58	6	Secretory	PlasmaMembrane	PlasmaMembrane
21	AqcTIP2-2	251	152.58	29.14	6		PlasmaMembrane	Vacoule
22	AqcTIP3-1	256	134.16	25.37	6		PlasmaMembrane	PlasmaMembrane
23	AqcTIP4-1	248	138.49	30.41	6		PlasmaMembrane	Vacoule
24	AqcTIP4-2	233	129.69	30.54	6		PlasmaMembrane	Vacoule
25	AqcTIP5-1	275	132.16	19.14	6		PlasmaMembrane	PlasmaMembrane
26	AqcXIP1-2	308	150.74	0	7		PlasmaMembrane	PlasmaMembrane
27	AqcXIP1-1	304	148.85	0.075	7		PlasmaMembrane	PlasmaMembrane
28	AqcXIP1-4	300	140.38	1.07	6		PlasmaMembrane	PlasmaMembrane
29	AqcXIP1-3	304	149.16	0.86	7		PlasmaMembrane	PlasmaMembrane

Footnote: Sequences showing less than six transmembrane domains predicted based on their sequence alignment. ExpAA: The expected number of amino acids in transmembrane helices. First60: The expected number of amino acids in transmembrane helices in the first 60 amino acids of the protein.

Table S3. Molecular weight and average PI of Aquilegia coerula Aquaporin.

Gene ID	Molecular weight (kDa)	Average PI
AqcNIP1-1	28.4	8.343
AqcNIP1-2	30	7.381
AqcNIP1-3	29.3	6.35
AqcNIP2-1	33.5	8.512
AqcNIP3-1	31.9	8.201
AqcNIP4-1	28.8	6.067
AqcNIP4-3	30.4	6.364
AqcNIP4-2	29.8	5.956
AqcNIP5-1	31.2	7.337
AqcPIP1-1	30.6	8.522
AqcPIP1-2	30.8	8.535
AqcPIP2-1	30.4	7.726
AqcPIP2-2	29.8	8.992
AqcSIP1-1	25.4	8.243
AqcSIP2-1	27.6	10.49
AqcTIP1-2	25.8	4.931
AqcTIP1-1	26.2	7.552
AqcTIP1-3	25.8	5.635
AqcTIP2-1	27.2	6.118
AqcTIP2-3	25	5.427
AqcTIP2-2	25.1	5.136
AqcTIP3-1	27	7.432
AqcTIP4-1	26	5.819
AqcTIP4-2	24.4	5.836
AqcTIP5-1	28.1	5.628
AqcXIP1-2	32.6	7.627
AqcXIP1-1	32.6	6.435
AqcXIP1-4	32	6.945
AqcXIP1-3	32.3	6.508

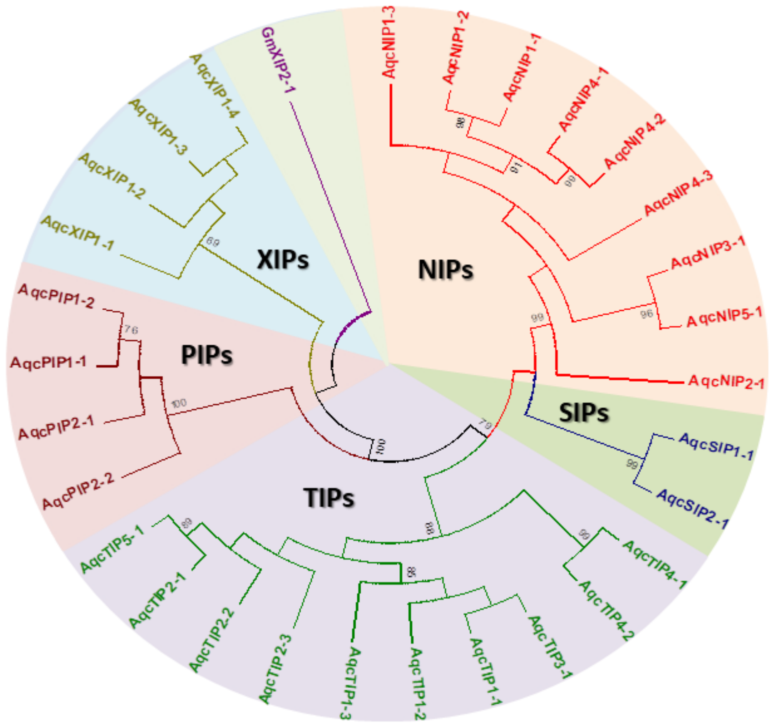

Figure S1. Phylogenetic analysis of *Aquilegia coerulea* aquaporins (AQPs). Phylogenetic tree showing distribution of aquaporins in five different subfamilies namely NOD26-like intrinsic proteins (NIPs), tonoplast intrinsic proteins (TIPs), plasma membrane intrinsic proteins (PIPs), small basic intrinsic proteins (SIPs) and uncharacterized intrinsic proteins (XIPs). The outgroup was taken from *Glycine max* (Gm).

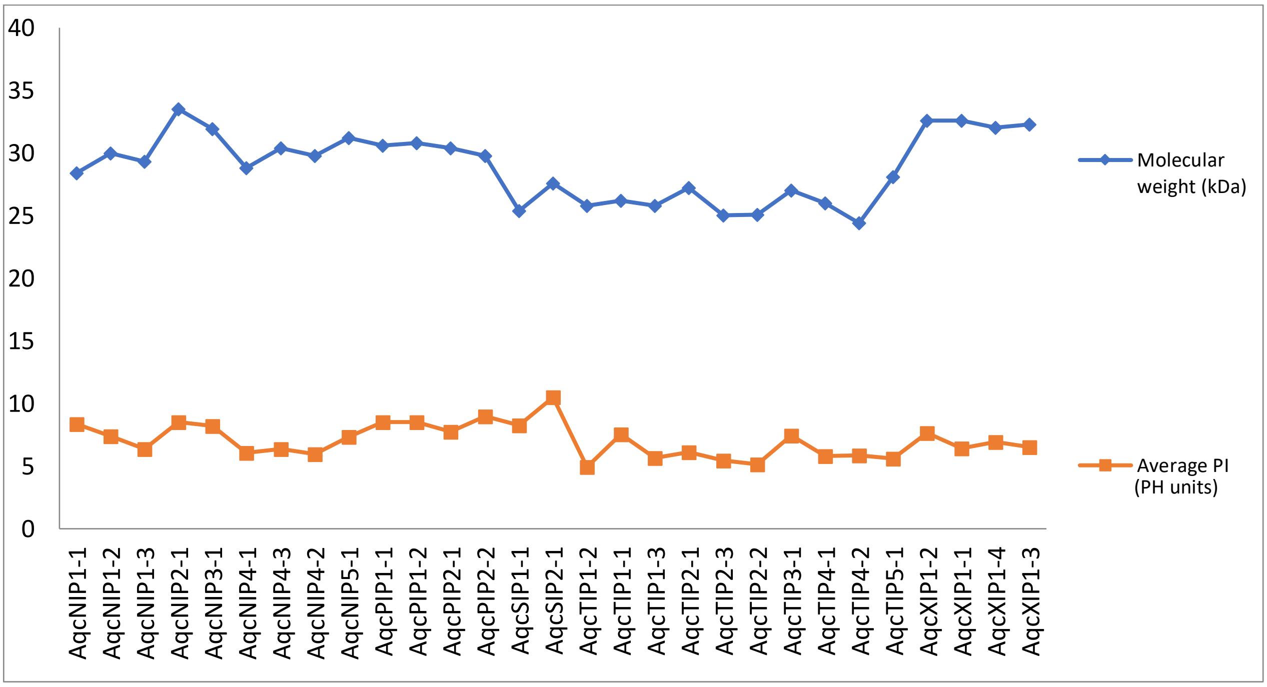

Figure S2. Molecular weight and isoelectric point of *Aquilegia coerulea* aquaporins (AQPs).

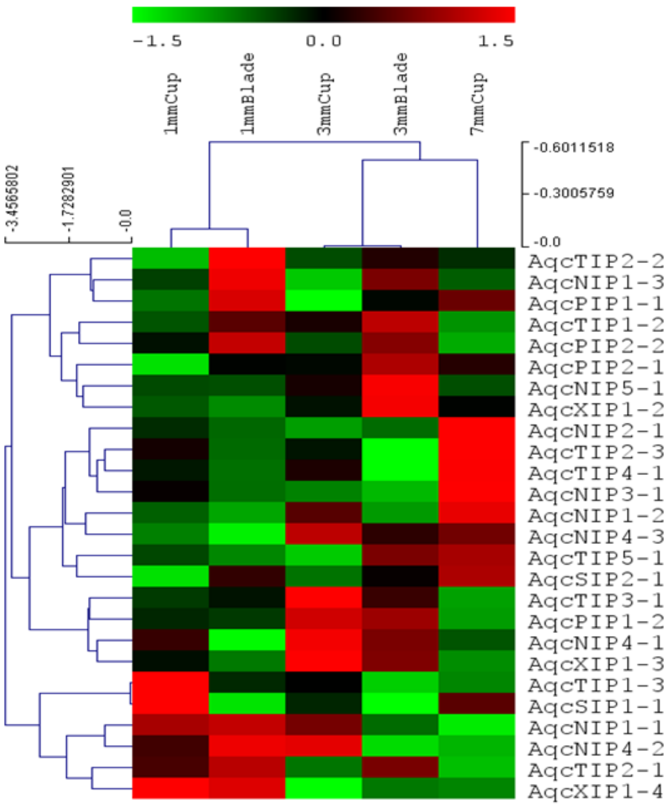

Figure S3. Heatmap showing expression profile of aquaporin genes in petal spur of *AquiIlegia coIerula*. Genes with no expression (0 FPKM values) were removed from the heatmap.
